# Overexpression of *plasmepsin II* and *plasmepsin III* does not directly cause reduction in *Plasmodium falciparum* sensitivity to artesunate, chloroquine and piperaquine

**DOI:** 10.1016/j.ijpddr.2018.11.004

**Published:** 2018-12-01

**Authors:** Duangkamon Loesbanluechai, Namfon Kotanan, Cristina de Cozar, Theerarat Kochakarn, Megan R. Ansbro, Kesinee Chotivanich, Nicholas J. White, Prapon Wilairat, Marcus C.S. Lee, Francisco Javier Gamo, Laura Maria Sanz, Thanat Chookajorn, Krittikorn Kümpornsin

**Affiliations:** a Genomics and Evolutionary Medicine Unit (GEM), Centre of Excellence in Malaria Research, Faculty of Tropical Medicine, Mahidol University, Bangkok, 10400, Thailand; b Molecular Medicine Program, Multidisciplinary Unit, Faculty of Science, Mahidol University, Bangkok, 10400, Thailand; c Tres Cantos Medicine Development Campus, GlaxoSmithKline, Parque Tecnológico de Madrid, Tres Cantos, 28760, Spain; d Laboratory of Malaria and Vector Research, National Institute of Allergy and Infectious Diseases, National Institutes of Health, Rockville, MD, 20852, USA; e Parasites and Microbes Programme, Wellcome Sanger Institute, Wellcome Genome Campus, Hinxton, CB10 1SA, United Kingdom; f Department of Clinical Tropical Medicine, Faculty of Tropical Medicine, Mahidol University, Bangkok, 10400, Thailand; g Mahidol-Oxford Tropical Medicine Research Unit, Faculty of Tropical Medicine, Mahidol University, Bangkok, 10400, Thailand; h Centre for Tropical Medicine and Global Health, Nuffield Department of Clinical Medicine, Churchill Hospital, Oxford, OX3 7LJ, United Kingdom; i Department of Biochemistry, Faculty of Science, Mahidol University, Bangkok, 10400, Thailand

**Keywords:** Artemisinin, Drug resistance, Malaria, Piperaquine, Plasmepsin

## Abstract

Artemisinin derivatives and their partner drugs in artemisinin combination therapies (ACTs) have played a pivotal role in global malaria mortality reduction during the last two decades. The loss of artemisinin efficacy due to evolving drug-resistant parasites could become a serious global health threat. Dihydroartemisinin-piperaquine is a well tolerated and generally highly effective ACT. The implementation of a partner drug in ACTs is critical in the control of emerging artemisinin resistance. Even though artemisinin is highly effective in parasite clearance, it is labile in the human body. A partner drug is necessary for killing the remaining parasites when the pulses of artemisinin have ceased. A population of *Plasmodium falciparum* parasites in Cambodia and adjacent countries has become resistant to piperaquine. Increased copy number of the genes encoding the haemoglobinases Plasmepsin II and Plasmepsin III has been linked with piperaquine resistance by genome-wide association studies and in clinical trials, leading to the use of increased *plasmepsin II*/*plasmepsin III* copy number as a molecular marker for piperaquine resistance. Here we demonstrate that overexpression of *plasmepsin II* and *plasmepsin III* in the 3D7 genetic background failed to change the susceptibility of *P. falciparum* to artemisinin, chloroquine and piperaquine by both a standard dose-response analysis and a piperaquine survival assay. Whilst *plasmepsin* copy number polymorphism is currently implemented as a molecular surveillance resistance marker, further studies to discover the molecular basis of piperaquine resistance and potential epistatic interactions are needed.

## Graphical abstract

**Figure:**
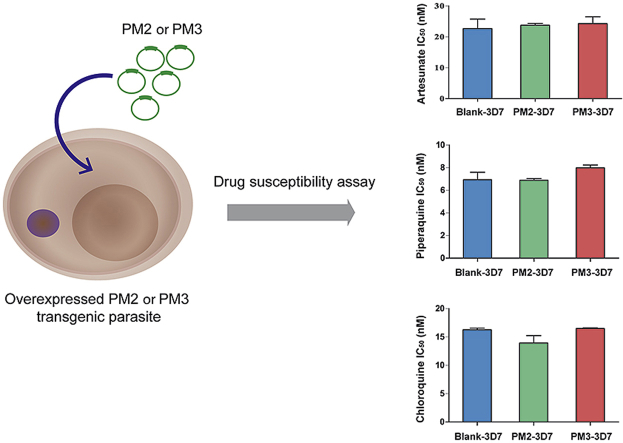

## 1 Introduction

Intraerythrocytic malaria parasites propagate successfully inside the human host by devouring a vast amount of haemoglobin. This is achieved by digesting the haemoglobin proteins by proteases in the food vacuole (Francis et al., 1997b). The digestive process employs a cascade of haemoglobinases cooperating to process various globins into short peptides (Klemba and Goldberg, 2002). The released haem molecules are not catabolised but instead are packed into relatively inert semicrystalline structures (haemozoin or malaria pigment) to prevent oxidative damage from iron-containing haem moieties (Sigala and Goldberg, 2014). The haemoglobinases work together as a large multi-subunit protein complex (Chugh et al., 2013). In *Plasmodium falciparum*, this haemoglobin-digestion complex is composed of three groups of proteases namely, falcilysin, falcipain and plasmepsin (Goldberg, 2005). The evolutionary history of these gene families reveals their specialized roles in the *Plasmodium* species as reflected by their respective independent gene expansion events (Ponsuwanna et al., 2016). This protein complex is associated with the actions of several antimalarial drugs including the quinoline related antimalarials (quinine, chloroquine, mefloquine etc) and artemisinin (Chugh et al., 2013). Genetic variations in these genes are also associated with changes in drug susceptibility (Amato et al., 2017; Klonis et al., 2011; Witkowski et al., 2017).

Recently, *P*. *falciparum* in Cambodia has started to lose sensitivity to piperaquine (Amaratunga et al., 2016; Chaorattanakawee et al., 2016), a key partner in one of the artemisinin combination therapy regimens. Piperaquine belongs to the 4-aminoquinoline chemical class, a group which includes chloroquine and amodiaquine (Pussard and Verdier, 1994). It retains activity against chloroquine resistant *P. falciparum* (Pascual et al., 2012). Piperaquine is a bisquinoline containing a bispiperazine propane connecting bridge (Raynes, 1999). Dihydroartemisinin-piperaquine combination therapy was implemented in Southeast Asia as an alternative to the artesunate-mefloquine regimen (Woodrow and White, 2017). Choosing the appropriate partner drug for artemisinin is an important public health issue since the life-saving artemisinin derivatives have short half-lives in the human body and require a partner drug with a considerably longer half-life to ensure complete parasite clearance and thus prevent the emergence and spread of drug resistance (Eastman and Fidock, 2009). Reduced artemisinin susceptibility leads to decreased parasite killing and a much greater residuum of parasites for the partner drug to remove, which increases the selective pressure on partner drug resistance.

Plasmepsins have become a topic of interest because of two independent genome-wide association studies (GWAS) showing variations in gene copy as a marker of piperaquine resistance (Amato et al., 2017; Witkowski et al., 2017). Plasmepsins belong to the aspartic protease group, which employs a catalytic dyad of two aspartic residues (Silva et al., 1996). There are ten Plasmepsin proteases in *P. falciparum* (Plasmepsin I to Plasmepsin X) (Ponsuwanna et al., 2016). Only Plasmepsin I - IV function in haemoglobin digestion, and the remaining enzymes have diverse biological functions (Ponsuwanna et al., 2016). For example, Plasmepsin V processes malarial proteins for transport from the intra-erythrocytic parasite to the red blood cell cytosol (Boddey et al., 2013; Sleebs et al., 2014). Interestingly, the genes encoding the haemoglobin-specific Plasmepsin proteins are located in the same gene cluster on chromosome 14 (Coombs et al., 2001). The expansion of the haemoglobin-specific plasmepsin genes occurred after the diversification of Plasmepsin functions (Ponsuwanna et al., 2016). Plasmepsin III is called Histo-Aspartic Protease (HAP) because its active site contains a dyad of histidine and aspartic acid residues instead of the two canonical aspartate residues (Bhaumik et al., 2009).

The amplification of the *plasmepsin II* and *III* genes is associated with reduced piperaquine sensitivity (Amato et al., 2017; Witkowski et al., 2017). A knock-out experiment of the genes encoding Plasmepsin II and Plasmepsin III in the 3D7 background slightly reduces the piperaquine IC_50_ values, but the effect is more pronounced in a survival assay with the *plasmepsin III* knockout parasite (Mukherjee et al., 2018). Emerging piperaquine resistance has the potential to be a global health threat since piperaquine containing antimalarial regimens are increasingly used in prevention and treatment. Thus, having a reliable molecular marker of resistance to this antimalarial drug is strategically important in the ongoing effort to contain the problem. However, it is also important to determine whether *plasmepsin* gene copy number polymorphism *per se* is directly responsible for piperaquine resistance since any genetic variation linked to certain phenotypes can be the results of either direct causation, fitness compensation or hitchhiking (Sackton and Hartl, 2016; Volkman et al., 2017). Identifying a molecular marker with tight linkage to the drug resistance phenotype is important for malarial surveillance and elimination efforts no matter what the mechanism underlying genetic linkage is. But the identification of causal mutations enables the design of strategies to counter resistance.

Here we tested whether the increased expression of *plasmepsin II* and *plasmepsin III* affects drug susceptibility phenotypes in *P. falciparum*. The levels of sensitivity to piperaquine, chloroquine and artesunate were determined using both a standard IC_50_ method and a survival assay. Significant changes in drug susceptibility were not observed in genetically engineered parasites. This observation suggests that increases in *plasmepsin II*/*III* gene copy number alone might not be causal to piperaquine resistance.

## 2 Material and methods

### 2.1 Overexpression of plasmepsin genes

The coding sequences of *plasmepsin II* (PF3D7_1408000) and *plasmepsin III* (PF3D7_1408100) genes were PCR amplified from genomic DNA of *Plasmodium falciparum* 3D7 using primer pairs containing restriction enzyme sites at 5′ and 3′ ends; PM2F 5′-ACAGCGGATCCATGGATATTACAGTAAGAGAACAT-3′ and PM2R 5′-CGCGCACCTAGGTTATAAATTCTTTTTAGCAAGAG-3´ (AvrII and SpeI give the same overhang site); PM3F 5′-CTTAGGATCCATGAATTTAACCATTAAAGA-3′ and PM3R 5′-CATCACTAGTTTATAAATTTTTGGCTAAAG-3′, respectively. The amplicon was cloned into the pBSD rep20 vector using the BamHI and SpeI sites (Sato et al., 2003; Wittayacom et al., 2010). The plasmid constructs were verified by DNA sequencing. The plasmid was transformed into the *P. falciparum* 3D7 strain using the DNA-loaded red blood cell method (Deitsch et al., 2001). Briefly, red blood cells were washed with cytomix (120 mM KCl, 0.15 mM CaCl_2_, 2 mM EGTA, 5 mM MgCl_2_, 10 mM K_2_HPO_4_/KH_2_PO_4_ and 25 mM HEPES, pH 7.6). The mixture of plasmid DNA and red blood cells was electroporated by using Bio-rad Gene Pulser (0.31 kV and capacitance 960 μFD). The loaded red blood cells were added into the culture with fresh schizont-infected red blood cells. The parasites were maintained in standard complete medium (RPMI 1640, 25 mM HEPES, 0.2% NaHCO_3_, 0.5% albumax, 146.9 μM hypoxanthine and 25 μg/ml gentamicin) under 2 μg/ml blasticidin (Gibco) selection, and emerging parasites were checked for the plasmid by PCR amplification using the *bsd* gene with primers bsd-F 5′-AGGTGCTTCTCGATCTGCAT-3′ bsd-R 5′-CATAACCAGAGGGCAGCAAT-3′ (Mamoun et al., 1999).

The RNA expression of *plasmepsin II* and *plasmepsin III* in transgenic parasites was analyzed by using RT-qPCR. The parasite lines were tightly synchronized by two consecutive rounds of 5% sorbitol treatment. Trophozoites (24–32 h after reinvasion) were collected for RNA extraction. *plasmepsin II*, *plasmepsin III* and *calmodulin* (PF3D7_1434200, the promoter driving the expression) are all highly expressed in this window (Otto et al., 2010). Every RT-qPCR reaction was performed at least in duplicate in two independent experiments. The raw cycle threshold (Ct) values of PM 1, PM 2, PM 3 and PM 4 with the no reverse transcriptase control and fold change calculation are shown in Supplementary Table 1. Total RNA was extracted by using Trizol reagent (Ambion) and sequentially treated with RNase-free DNase I (Promega). Superscript III First-Strand Synthesis System (Invitrogen) was used to convert RNA to cDNA with random hexamer primers (Invitrogen). qPCR reactions were performed using KAPA SYBR^®^ Fast qPCR kit master mix, according to the manufacturer's recommended conditions at 95 °C for 3 s, 55 °C for 20 s and 72 °C for 20 s (35 cycles) with the Mastercycler^®^ ep realplex (Eppendrof). Four plasmepsin genes, PM 1 (PF3D7_1407900), PM 2, PM 3 and PM 4 (PF3D7_1407800), were quantified by comparing with Actin I (PF3D7_1246200), an endogenous control gene in *P. falciparum* 3D7 (blank vector control) and *P. falciparum* 3D7 overexpressed PM 2 and overexpressed PM 3 transgenic parasites. The primer information is as follows: PM1-F 5ʹ-AGGTAATGCTGGTGATAGTGTAA-3ʹ, PM1-R 5ʹ-CATTGAGCACTTGGAACCCA-3ʹ, PM2-F 5ʹ-TGGTGATGCAGAAGTTGGAG-3ʹ, PM2-R 5ʹ-TCCTGCAGTTGTACATTTAACAC-3ʹ, PM3-F 5ʹ-AGAATCCTTTAACACGTTTCGAG-3ʹ, PM3-R 5ʹ-ACCTCCTGCCAAAACTATGAA-3ʹ, PM4-F 5ʹ-TGGCTCTTACCGTTAAAGAAGA-3ʹ, PM4-R 5ʹ-CACACCCGTAATAAGGACAAACA-3ʹ, Actin I—F 5′-AGCAGCAGGAATCCAC ACA-3′ and Actin I-R 5′-GATGGTGCAAGGGTTGTAA-3´. The fold change of gene expression was calculated by using the 2^−ΔΔCt^ method (Livak and Schmittgen, 2001; Schmittgen and Livak, 2008). Controls with no reverse transcriptase were included to monitor genomic DNA contamination. Student's t-test was used for statistical testing.

Western analysis was performed with the parasite extract prepared from 3% synchronous trophozoites. Parasitised red blood cells were lysed with 0.15% saponin on ice for 1 min. The parasite pellets were washed twice with Phosphate Buffer Saline (PBS) containing protease inhibitor cocktail cOmplete™ (Roche). The parasite pellets were heated in NuPAGE LDS sample buffer (Thermo Fisher Scientific) and were analyzed in 4–12% Bis-Tris NuPAGE Gel (Thermo Fisher Scientific). The protein was transferred onto Hybond^®^ ECL™ nitrocellulose membranes (GE Healthcare) and subsequently blocked with 5% milk. The blots were incubated with 1:3000 polyclonal anti-plasmepsin II (1:3000) and 3F10-6 anti-plasmepsin III (1:10,000) provided by MR4, with anti-ERD2 polyclonal antibody (1:2000) as a control (Francis et al., 1997a; Liu et al., 2005). The secondary antibodies used for detection were goat anti-rabbit IgG HRP (GE Healthcare) and goat anti-mouse HRP (Abcam). Amersham ECL prime Western blotting detection reagent (GE Healthcare) was employed to visualise the signal. The protein bands were analyzed by Image Studio Lite (LI-COR).

### 2.2 Piperaquine and chloroquine sensitivity assays

Piperaquine was kindly provided by WWARN. Chloroquine was purchased from Fluka Analytical (PHR1258). Piperaquine and chloroquine were prepared in 0.5% lactic acid (Sigma) and sterile deionized water, respectively. Synchronous parasites at the ring stage were adjusted to 0.5% parasitemia in 4% hematocrit. The drugs were prepared by two-fold serial drug dilutions in the 96-well plate. The drug concentrations of chloroquine and piperaquine were varied at 500 nM–0.98 nM and 100 nM - 0.09 nM, respectively. The plates were incubated at 37 °C for 72 h, and parasite growth after drug treatment was measured using SYBR Green I (Invitrogen) in lysis buffer (20 mM Tris-HCl, 5 mM EDTA, 0.008% Saponin and 0.08% Triton X). The reaction was incubated for 1 h in the dark at room temperature, and the fluorescence intensity was determined with excitation and emission wavelength at 490/540 nm. Parasite survival was calculated from sample (with drug) and parasite growth control (without drug), and the signal was normalized with the background signal (the highest concentration of each drug). The dose-response assay was performed for all three parasite lines in duplicate in three independent experiments, and the dose-response curves for IC_50_ and IC_90_ determination were plotted by using GraphPad Prism 6. Student's t-test was used for statistical testing.

*In vitro* piperaquine survival assay (PSA) was performed in *P. falciparum* 3D7 and PM 2 and PM 3 overexpression parasites. PSA was performed as previously described (Duru et al., 2015). Briefly, early stage parasites were prepared by using 70% Percoll (GE Healthcare) and 5% sorbitol treatment. Early ring stage parasites at 0–3 h post invasion were split to culture in standard complete medium containing 200 nM piperaquine and in drug-free medium for 48 h. The parasites were washed with incomplete media and further incubated for additional 24 h in complete medium. The thin blood smears from the experiments were stained with Giemsa, and the parasites were counted for 20,000 erythrocytes. The percent PSA survival rate was calculated by using 100 x (number of viable parasites in exposed culture/number of viable parasites in non-exposed culture) (Duru et al., 2015).

The bimodal shape of the piperaquine dose-response curve was recently reported as a phenotype for piperaquine resistance (Bopp et al., 2018). The same protocol was followed in this study. Briefly, the early ring stage parasites were obtained using Percoll and sorbitol synchronisation. Piperaquine (Sigma-Aldrich) was dissolved in 0.5% lactic acid and 0.1% Triton X-100. The twenty-four piperaquine concentrations (two-fold dilution) were prepared on an assay plate using D300e digital dispenser (Tecan) with the highest drug concentration at 100 μM. The culture at 1% parasitemia in 1% hematocrit was incubated with the drug for 72 h, and parasite growth was determined by using SYBR Green I. Each experiment was done in duplicate for each parasite line. Three biological replicates were performed.

### 2.3 Artemisinin sensitivity assay

The parasites at the early ring stage (0–6 h post invasion) were prepared by centrifugation at 70% Percoll and subsequently treated with 5% sorbitol (approximately 5 h after Percoll synchronisation). Artesunate was purchased from Guilin Pharmaceutical (Guangxi, China) and was dissolved in 5% sodium bicarbonate. Artesunate was prepared by two-fold serial dilutions in 96-well plate. The drug concentrations were varied (1 μM-1 nM). The IC_50_ determination for artesunate was performed by using a newly modified method based on the Trophozoite Maturation Inhibition assay (TMI) (Chotivanich et al., 2014). Lactate dehydrogenase-based Inhibitory Concentration Assay (LICA) was developed in order to reduce the subjective read-out in determining live and dead parasites by microscopy after artesunate treatment. The assay was validated by comparing with the original TMI method. To optimize the assay read-out, drug exposure time in LICA was varied from 1, 3 and 5 h. *Plasmodium falciparum* lactate dehydrogenase activity from parasite lysate was determined to represent parasite viability after 48 h of initial drug treatment instead of using Giemsa staining. The parasite survival measured by using TMI and LICA was compared using Pearson's correlation.

The early ring stage parasites were adjusted to 1% parasitemia in 2% hematocrit and incubated with artesunate for 3 h. The drug was aspirated and replaced with drug-free complete medium. The plates were incubated for an additional 48 h and kept at −80 °C. Parasite growth was determined by measuring LDH activity (Gamo et al., 2010). The culture plates were thawed at room temperature and mixed with LDH reaction mixture, consisting of 100 mM sodium L-lactate, 100 μM 3-acetylpyridine adenine dinucleotide (APAD), 125 μM nitroblue tetrazolium **(**NBT), 200 μg/ml diaphorase, 0.6% Tween 20, and 35 mM Tris-HCl pH 8.0. The reaction was conducted at room temperature for 30 min in the 96-well half-area clear plate. The signal was determined by optical density at 650 nm (OD_650_).

Percent parasite survival was calculated based on the LDH signal at OD_650_ of the sample (with drug) compared to the parasite growth control (without drug). The signal was normalized with the background signal (the highest concentration of artesunate). The dose-response curve for IC_50_ determination was plotted by using GraphPad Prism 6, and Student's t-test was used for statistical testing.

## 3 Results

### 3.1 Plasmepsin overexpression in *P. falciparum*

**Fig. 1:**
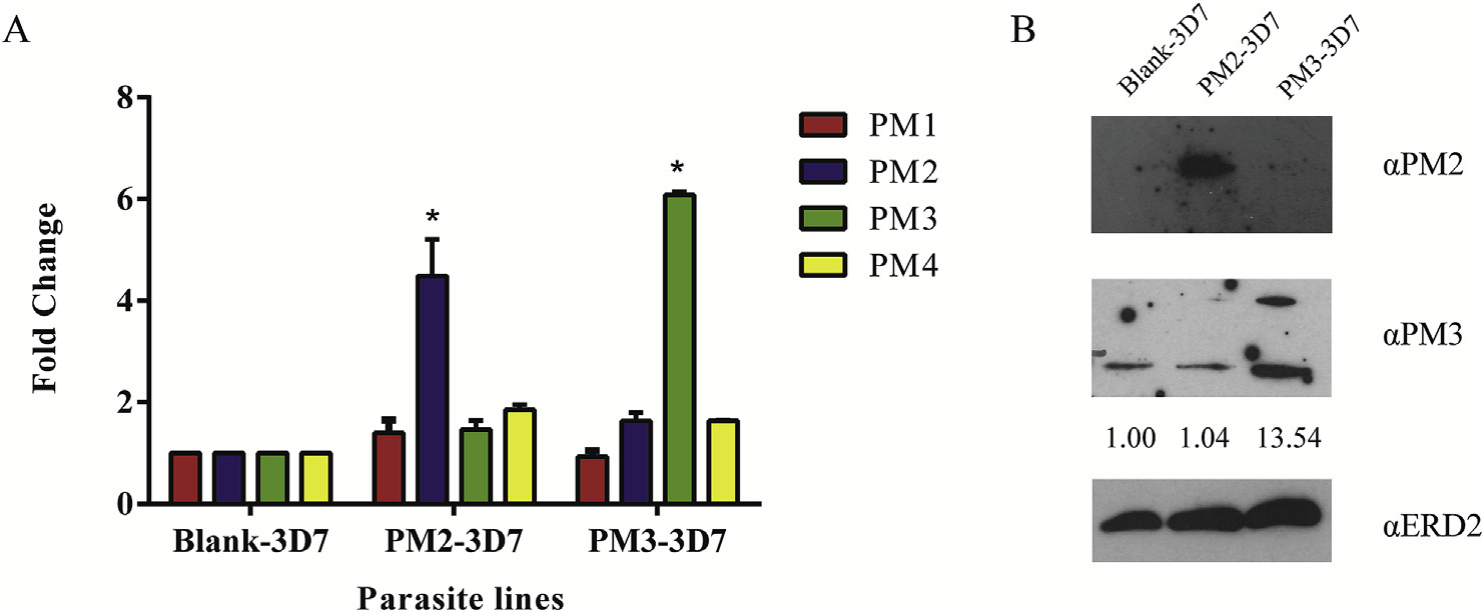
Overexpression of *plasmepsin* genes. (A) Three transgenic lines were generated for this study with the 3D7 parasites transfected with a blank vector, Plasmepsin II vector or Plasmepsin III vector, designated as Blank-3D7, PM2-3D7 and PM3-3D7, respectively. The increase in the gene expression level was presented as fold change. The level of expression of plasmepsin 1-4 was monitored by RT-qPCR (shown as PM1 to PM4). The fold change values were compared to those of Blank-3D7, giving the fold change value of 1 for the controls. The fold change values of PM2 and PM3 expression were significantly increased in PM2-3D7 and PM3-3D7, respectively (*p-value < 0.05). (B) Western analysis of Plasmepsin proteins. The extract from synchronized trophozoite-stage parasites was used for the analysis and probed with the antibodies against PM2 (α-PM2) and PM3 (α-PM3) with the antibodies against ERD2 (α-ERD2) as a loading control. Signal quantification with Image Studio Lite was normalized with the ERD2 control and presented as fold increase in comparison to Blank-3D7 (shown as numbers under the α-PM3 panel). The fold increase value for PM2 was not available since the PM2 band was not visible in Blank-3D7 even after extended exposure time.

*P. falciparum* 3D7 parasites were transfected with malarial expression vectors containing *plasmepsin II* and *plasmepsin III* with the empty vector as a negative control (designated PM2-3D7, PM3-3D7 and Blank-3D7, respectively). Expression of the respective genes is driven by a *P. falciparum* calmodulin promoter (Crabb and Cowman, 1996; Wittayacom et al., 2010). The increase in the RNA level was consistently observed using RT-qPCR (Fig. 1). The *P. falciparum* selectable marker for the episomal expression vector is blasticidin S deaminase (*bsd*). At 2 μg/ml blasticidin, the level of expression of *plasmepsin II* and *plasmepsin III* transcripts at the trophozoite stages was increased significantly in PM2-3D7 and PM3-3D7 respectively (Fig. 1A). The increases in the transcript levels exceeded the fold changes observed in the field samples (Witkowski et al., 2017). The amount of respective plasmepsin protein was also increased as observed by Western blot analysis (Fig. 1B).

**Fig. 2:**
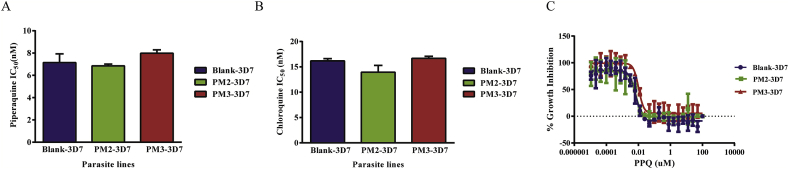
Effect of *plasmepsin* overexpression on piperaquine and chloroquine susceptibility. The IC_50_ values for piperaquine (A) and chloroquine (B) in the transgenic lines were determined. The information is reported in Table 1, and the dose-response curves are shown in Supplementary Fig. 1. (C) Dose-response curves at high piperaquine concentrations. The dose-response curve showing the bimodal pattern was not found even when the drug concentrations and the experiments were done according to the previous report presenting the bimodal pattern (Bopp et al., 2018).

The parasites were tested for their susceptibility to piperaquine and chloroquine. Chloroquine was included in the experiment because of its structural similarity to piperaquine and its clinical use for treating *Plasmodium vivax* and other non-*P. falciparum* malaria infections in Southeast Asia. We did not observe a significant change in the levels of piperaquine and chloroquine resistance in the transgenic parasite lines (Fig. 2 and Table 1). Assessment of piperaquine susceptibility was also determined using a piperaquine survival assay (PSA) (Duru et al., 2015). This assay relies on the exposure of the parasites (0–3 h post invasion) to 200 nM piperaquine for 48 h before removing the drug and subsequent monitoring of surviving parasites microscopically after an additional 24 h. Similarly, overexpression of *plasmepsin II* and *plasmepsin III* did not increase the number of surviving parasite using the PSA method after piperaquine exposure (Table 1). Recently, a bimodal dose-response curve was proposed to be an alternative readout for piperaquine resistance (Bopp et al., 2018). We followed the same protocol to determine whether a second peak of parasites surviving piperaquine exposure could be observed in the parasite lines overexpressing *plasmepsin II* and *plasmepsin III* (Fig. 2C). In this genetic background, the second peak was not present even when the concentration was extended to 100 μM (Fig. 2C). Thus, genetically engineered overexpression of *plasmepsins II* and *III* has no apparent effect on parasite sensitivity to piperaquine and chloroquine.

**Table 1:** Drug susceptibility level in the parasites over-expressing *plasmepsin II* and *plasmepsin III*.

Parasite line	Piperaquine				Chloroquine				Artesunate				PSA Survival rate (number of total parasites found)
	IC_50_		IC_90_		IC_50_		IC_90_		IC_50_		IC_90_		
	Mean ± SD, nM	*p-*value	Mean ± SD, nM	*p-*value	Mean ± SD, nM	*p*-value	Mean ± SD, nM	*p*-value	Mean ± SD, nM	*p*-value	Mean ± SD, nM	*p*-value	
Blank-3D7	7.17 ± 0.77		9.25 ± 1.16		16.27 ± 0.29		22.33 ± 1.49		26.1 ± 0.05		56.34 ± 0.49		0% (of 897 counts)
PM2-3D7	6.86 ± 0.16	0.7716	9.48 ± 0.97	0.8958	13.94 ± 1.3	0.3136	22.49 ± 3.67	0.9739	23.3 ± 0.6	0.1323	59.52 ± 5.09	0.4063	0% (of 1419 counts)
PM3-3D7	7.97 ± 0.18	0.4741	11.29 ± 0.15	0.3261	16.49 ± 0.11	0.59	21.04 ± 2.47	0.7052	27.51 ± 1.47	0.5131	69.47 ± 9.88	0.4101	0% (of 1897 counts)

PSA survival rate (%) = (Number of viable parasites in exposed culture/Number of viable parasites in non-exposed culture) x 100; the PSA value above ≥10% is considered piperaquine resistance (Duru et al., 2015).

### 3.2 Effect of plasmepsin overexpression on artemisinin sensitivity

**Fig. 3:**
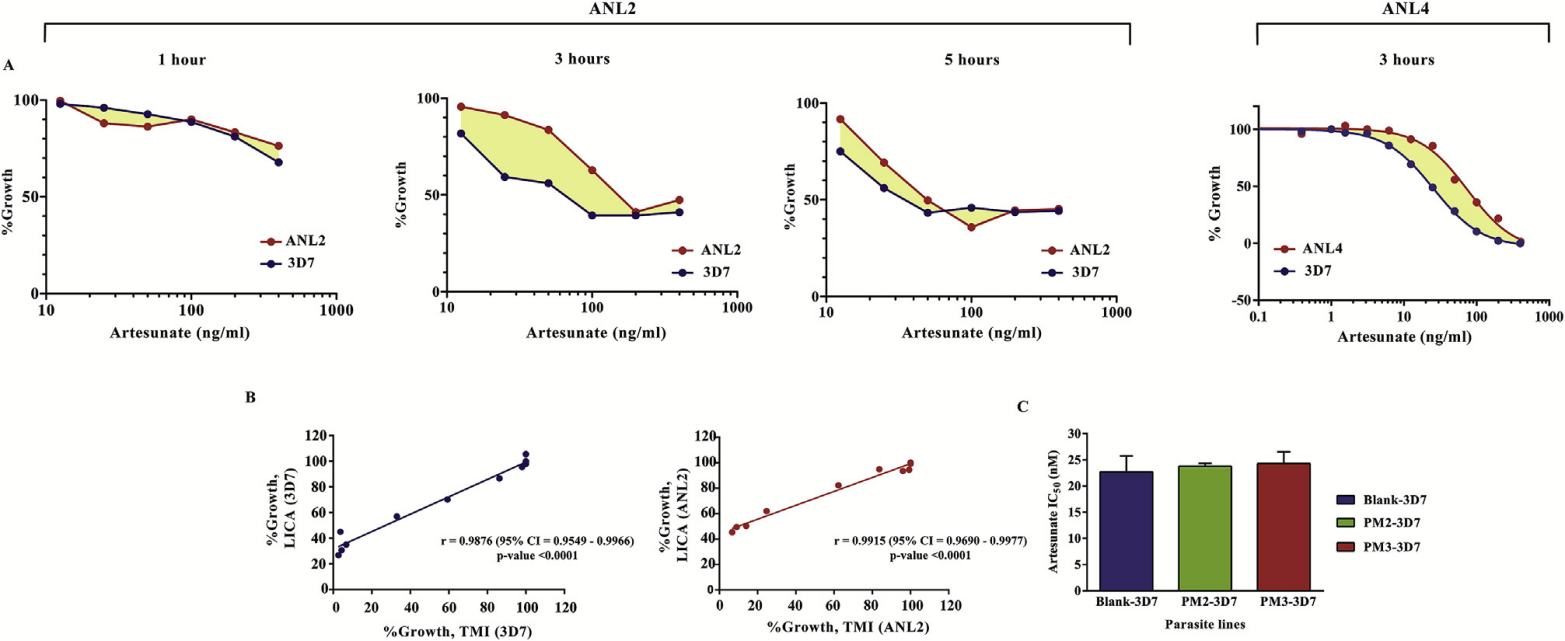
Effect of *plasmepsin* overexpression on artemisinin susceptibility. (A) Effect of drug exposure time on LICA. The graphs represent the difference in percent survival at specific drug concentrations. The drug exposure times were set at 1, 3 and 5 h. The difference between sensitive and resistant strains is maximized at the drug exposure time of 3 h. A short exposure time (1 h) is not enough to differentiate between strains. Longer exposure time (5 h) affected the ability to differentiate between sensitive and resistant strains. (B) Strong correlation between the artemisinin susceptibility values obtained from TMI and LICA. Pearson's correlation coefficients between methods are statistically significant in the assays performed with the 3D7 and ANL2 *P. falciparum* lines. (C) Effect of *plasmepsin* overexpression on artesunate susceptibility. The artesunate susceptibility IC_50_ values were determined in the three transgenic lines without any significant difference. Each experiment was done in duplicate. Two independent biological parasite samples were tested. The information is reported in Table 1 and the dose-response curves are shown in Supplementary Fig. 1.

Artemisinin and its derivatives are widely used throughout the tropical world. We wanted to test the effect of *plasmepsin* overexpression on artemisinin susceptibility. Reduced artemisinin sensitivity was reported in Cambodian patients manifesting as delayed parasite clearance following artemisinin treatment (Ashley et al., 2014; Dondorp et al., 2009). Nevertheless, the level of resistance might not have reached the clinical definition of drug resistance because the drug can still achieve a certain degree of parasite clearance. A shift in *in vitro* artemisinin susceptibility is also relatively small (Chotivanich et al., 2014). These parasites confer artemisinin resistance during the first 3–6 h of their erythrocytic life cycle. Even though the time window of resistance is relatively small considering the 48-h erythrocytic cycle, it allows a significant portion of circulating ring stage parasites to withstand a pulse of labile artemisinin (Woodrow et al., 2005). This leads to the development of the Ring Survival Assay protocol (RSA) to assess artemisinin sensitivity (Witkowski et al., 2013). In RSA, the early ring parasites are exposed to a pulse of artemisinin for only 6 h, and their survival is determined after 66 h in the absence of artemisinin to determine “Percent Survival”. This method has become a useful tool in tracking these parasites, but the Percent Survival readout does not provide a definite value of artemisinin sensitivity. An alternative method called the Trophozoite Maturation Inhibition assay (TMI) measures the ability of early ring parasites to mature into trophozoites under exposure to different artemisinin concentrations and provides IC_50_ values (Chotivanich et al., 2014). However, it is demanding as it relies on tedious microscopic visualisation to assess the parasite development at every drug concentration. A robust and simple method for determining artemisinin sensitivity was developed for this study based on colourimetric measurement of *P. falciparum* lactate dehydrogenase, called the Lactate dehydrogenase-based Inhibitory Concentration Assay (LICA). This LICA method allows a rapid measurement of artemisinin IC_50_ in *P. falciparum*. Since artemisinin resistance is manifested only in the early ring stage, 0-6 h-post-invasion parasites were used for subsequent drug-sensitivity assays. Malaria cultures containing 1% early ring parasites were maintained in 96-well plates with a range of artesunate concentrations ranging from 0.5 nM to 1 μM. Drug-containing medium was removed after a 3-h exposure, which maximized the difference between sensitive and resistant parasites (Fig. 3A). The detail of the parasites and their genetic compositions are previously described (Bunditvorapoom et al., 2018; Ponsuwanna et al., 2016). ANL2 (*kelch 13* wild-type) and ANL4 (*kelch 13* C580Y) came from the clinical cases with delayed clearance following artemisinin treatment (Bunditvorapoom et al., 2018). To differentiate between signal output from dead and living parasites, the culture was continued in drug-free medium for another intra-erythrocytic asexual cycle to increase the numbers of live parasites since only the surviving parasites can propagate further. *P. falciparum* lactate dehydrogenase was used as a colourimetric readout. IC_50_ values using LICA and TMI assays were comparable (Fig. 3B) and were also consistent with the data from the previously published values using the TMI method (Chotivanich et al., 2014). We then tested the effect of plasmepsin overexpression on artemisinin susceptibility. There was no significant shift in their susceptibility to artesunate (Fig. 3C and Table 1).

## 4 Discussion

Here we present the evidence that the overexpression of *plasmepsin II* and *plasmepsin III* alone does not affect *P. falciparum* susceptibility to artesunate, chloroquine or piperaquine. Parasites from Cambodia have developed resistance to piperaquine, a key partner drug to artemisinin (Amaratunga et al., 2016; Chaorattanakawee et al., 2016). Copy number polymorphism at the genes encoding Plasmepsin II and Plasmepsin III is associated with piperaquine resistance (Amato et al., 2017; Witkowski et al., 2017). This led to the use of their copy numbers as a molecular marker for piperaquine resistance (Imwong et al., 2017). Our findings suggest that gene amplification of *plasmepsin II* and *III* is not the causal mutation responsible for piperaquine resistance. Thus, it is important for the malaria research field to consider other possible causes underlying piperaquine resistance.

One of the leading candidates is mutations at the gene encoding *Plasmodium falciparum* chloroquine resistance transporter (PfCRT) (Wellems and Plowe, 2001). The K76T mutation was originally mapped and functionally proven to be a causal mutation for chloroquine resistance (Fidock et al., 2000). Chloroquine and piperaquine share the same 4-aminoquinoline scaffold. It is reasonable to expect that *pfcrt* mutations could affect parasite sensitivity to piperaquine. In fact, a F145I mutation in PfCRT was shown to be associated with piperaquine resistance especially in the background with *plasmepsin* copy number polymorphism (Agrawal et al., 2017). Furthermore, a C101F PfCRT mutation was identified during piperaquine selection (Dhingra et al., 2017). This mutation, when introduced into a *P*. *falciparum* Dd2 background, significantly increases IC_90_ values to piperaquine by more than two orders of magnitude (Dhingra et al., 2017). A recent study identified additional PfCRT mutations that have increased in prevalence in Cambodian parasites and confer reduced piperaquine susceptibility when introduced into Dd2 parasites by genome editing (Ross et al., 2018). The reduction in piperaquine susceptibility in transgenic experiments and the GWAS data strongly support the role of PfCRT variant(s) in piperaquine resistance.

It is undeniable that the parasites with *plasmepsin* copy number polymorphism are spreading in Cambodia (Amato et al., 2018; Imwong et al., 2017). This level of selective pressure could imply the vital role of plasmepsin gene amplification in providing certain benefits to the parasites. The data presented here provide an experimental proof that introducing extra amounts of Plasmepsin II and Plasmepsin III does not reduce piperaquine susceptibility by itself in the 3D7 parasite genetic background. Based on the IC_50_ data, it also does not affect chloroquine and artesunate susceptibilities. It is possible that *plasmepsin II*/*plasmepsin III* gene copy number polymorphism could help with fitness compensation in drug resistance evolution, similar to *gch1* amplification in pyrimethamine resistance (Kumpornsin et al., 2014a, 2014b). Further studies on the effect of *plasmepsin* copy number polymorphism in various genetic backgrounds are necessary because genetic interactions between plasmepsin and other genetic variants might be the underlying driver of piperaquine resistance selection.

The use of molecular markers in drug resistance surveillance is an essential component of any malaria control programme. However, it is prudent to weigh the scientific significance of each molecular tool. The role of each malarial drug resistance marker should be exhaustively validated, and any adoption of a drug policy change based on poorly defined molecular markers should be avoided at all cost.

## Appendix A Supplementary data

Supplementary Fig. 1 Supplementary Fig. 1

Data Profile Data Profile
