# Supplementary material for: Overexpression of plasmepsin II and plasmepsin III does not directly cause reduction in Plasmodium falciparum sensitivity to artesunate, chloroquine and piperaquine
Source: Int J Parasitol Drugs Drug Resist. 2018 Dec 1;9:16–22. doi: 10.1016/j.ijpddr.2018.11.004 (PMC6304341; doi:10.1016/j.ijpddr.2018.11.004)
Supplement: Supplementary Fig. 1 [file mmc2.doc]

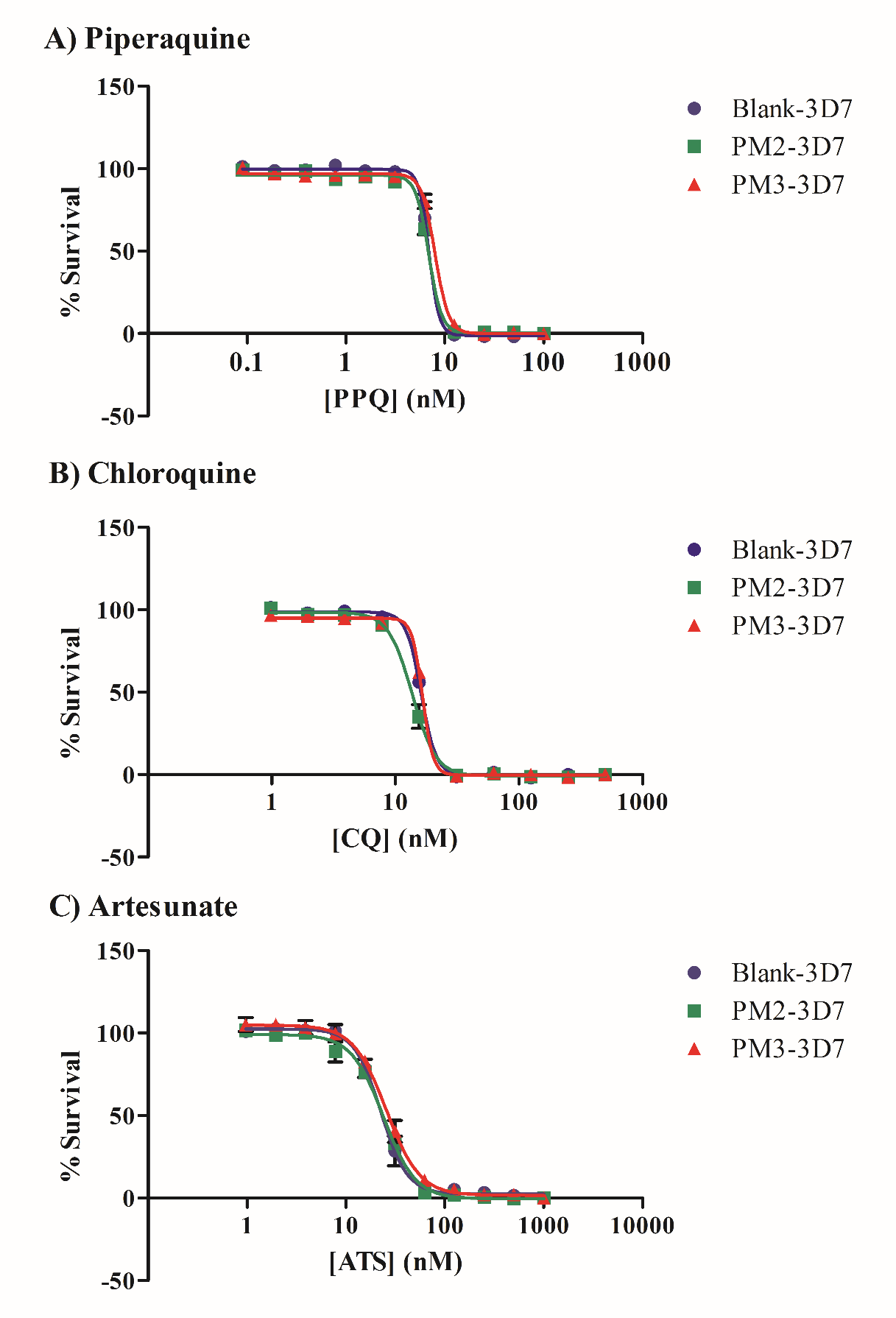


Supplementary Figure 1. Dose-response analyses of the transgenic *P. falciparum* parasites. The dose-response curves of piperaquine (A), chloroquine (B) and artesunate (C) in three transgenic lines were plotted using GraphPad Prism.
